# Synthesis and In Vitro Study of Antiviral Activity of Glycyrrhizin Nicotinate Derivatives against HIV-1 Pseudoviruses and SARS-CoV-2 Viruses

**DOI:** 10.3390/molecules27010295

**Published:** 2022-01-04

**Authors:** Vladislav V. Fomenko, Nadezhda B. Rudometova, Olga I. Yarovaya, Artem D. Rogachev, Anastasia A. Fando, Anna V. Zaykovskaya, Nina I. Komarova, Dmitry N. Shcherbakov, Oleg V. Pyankov, Andrey G. Pokrovsky, Larisa I. Karpenko, Rinat A. Maksyutov, Nariman F. Salakhutdinov

**Affiliations:** 1 Department of Medicinal Chemistry, N. N. Vorozhtsov Novosibirsk Institute of Organic Chemistry, Siberian Branch of the Russian Academy of Sciences, Lavrentiev Ave. 9, 630090 Novosibirsk, Russia; fomenko@nioch.nsc.ru (V.V.F.); artrogachev@yandex.ru (A.D.R.); komar@nioch.nsc.ru (N.I.K.); anvar@nioch.nsc.ru (N.F.S.); 2 State Research Center of Virology and Biotechnology VECTOR, Rospotrebnadzor, 630559 Koltsovo, Russia; nadenkaand100@mail.ru (N.B.R.); zaykovskaya_av@vector.nsc.ru (A.V.Z.); dnshcherbakov@gmail.com (D.N.S.); pyankov_ov@vector.nsc.ru (O.V.P.); lkarpenko@ngs.ru (L.I.K.); maksyutov_ra@vector.nsc.ru (R.A.M.); 3 Zelman Institute for Medicine and Psychology, Novosibirsk State University, Pirogov Str. 1, 630090 Novosibirsk, Russia; nastyafando@gmail.com (A.A.F.); agpok@inbox.ru (A.G.P.)

**Keywords:** human immunodeficiency virus type 1, SARS-CoV-2, entry inhibitors, nicotinates of glycyrrhizic acid

## Abstract

When developing drugs against SARS-CoV-2, it is important to consider the characteristics of patients with different co-morbidities. People infected with HIV-1 are a particularly vulnerable group, as they may be at a higher risk than the general population of contracting COVID-19 with clinical complications. For such patients, drugs with a broad spectrum of antiviral activity are of paramount importance. Glycyrrhizinic acid (Glyc) and its derivatives are promising biologically active compounds for the development of such broad-spectrum antiviral agents. In this work, derivatives of Glyc obtained by acylation with nicotinic acid were investigated. The resulting preparation, Glycyvir, is a multi-component mixture containing mainly mono-, di-, tri- and tetranicotinates. The composition of Glycyvir was characterized by HPLC-MS/MS and its toxicity assessed in cell culture. Antiviral activity against three strains of SARS-CoV-2 was tested in vitro on Vero E6 cells by MTT assay. Glycyvir was shown to inhibit SARS-CoV-2 replication in vitro (IC_50_2–8 μM) with an antiviral activity comparable to the control drug Remdesivir. In addition, Glycyvir exhibited marked inhibitory activity against HIV pseudoviruses of subtypes B, A6 and the recombinant form CRF63_02A (IC_50_ range 3.9–27.5 µM). The time-dependence of Glycyvir inhibitory activity on HIV pseudovirus infection of TZM-bl cells suggested that the compound interfered with virus entry into the target cell. Glycyvir is a promising candidate as an agent with low toxicity and a broad spectrum of antiviral action.

## 1. Introduction

In the spring of 2020, the world was confronted with a fundamentally new type of threat—a global pandemic of the coronavirus infection COVID-19. Although it is not the first pandemic in human history, in some respects it differs significantly from all previous pandemics, in particular in its global impact on the economy and the politics of every country in the world. According to official figures, the economic damage caused by COVID-19 is greater than that caused by all other infectious diseases combined. The development of vaccines and effective therapeutics is a major undertaking of the global scientific community, at an unprecedented scale. Although effective vaccines against SARS-CoV-2 are available, they are likely to become less effective with the emergence of new mutant variants, prompting new vaccine development efforts. Progress in the identification of effective therapeutics has been much more modest, with no drug universally recognized as effective against SARS-CoV-2.

In the quest for effective therapies against COVID-19, it is necessary to take into account the fact that there are categories of people with serious co-morbidities. One vulnerable group comprises people infected with HIV-1 (human immunodeficiency virus). HIV-positive people, especially those at advanced stages of infection and not receiving antiretroviral therapy, are at a higher risk of contracting opportunistic infections and developing AIDS-related complications [1]. They are also at a higher risk of contracting SARS-CoV-2 and developing clinical complications of COVID-19 than the general population [2]. There is an urgent, unmet medical need for new drugs with a broad spectrum of antiviral activity.

Antiretroviral drugs for the prevention of SARS-CoV-2 have been evaluated in several studies, often with conflicting results [3,4,5]. Thus, in one recent study, people with HIV receiving tenofovir disoproxil fumarate were found to be less likely to be infected with SARS-CoV-2. However, other studies indicate that tenofovir-based HIV pre-exposure prophylaxis does not protect against infection with the new coronavirus or mitigate the course of COVID 19 disease [6]. Attempts to identify drugs with antiviral activity against both HIV-1 and SARS-CoV-2 are ongoing.

The majority of FDA-approved antiviral compounds are small molecules. Most target the viral replicative machinery; and very few target host cells or cellular mechanisms. FDA-approved antiviral drugs may be grouped into different classes based on their structure and mechanisms of action: structural analogues, entry inhibitors, integrase inhibitors, nucleoside reverse transcriptase inhibitors, non-nucleoside reverse transcriptase inhibitors, protease inhibitors, inhibitors specific to certain viruses, interferons, immunomodulators, antimitotic inhibitors and oligonucleotides [7]. Importantly, these drugs may be prescribed either as mono- or combination therapy. In monotherapy, antiviral drugs target either the virus or the host, whereas in combination therapy, most drugs target viral components, and only a few target both viral and host proteins. The global and rapid spread of COVID-19 presents a unique challenge because drugs are needed to fight against both viral transmission and clinical manifestations of viral infections. Importantly, the strategy of repurposing medicines in the search for effective agents against the SARS-CoV-2 virus has failed [8].

Currently, HIV infections are mainly treated with antiretroviral therapy (ART), which aims to suppress viral replication, thus preserving the body′s immunological functions and reducing the risk of transmission of the virus [9]. However, the emergence of HIV resistance to ARTs reduces treatment efficacy and increases HIV/AIDS mortality [10]. The development of HIV-1 entry inhibitors is a promising new approach to address the emerging problem. Targeting the initial step of viral entry onto the target cell offers a number of advantages, compared to other steps in the HIV-1 life cycle [11]. Firstly, because the virus is prevented from having its genome integrated into that of the host, thus preventing the creation of latent virus reservoirs. Secondly, entry inhibitors do not need to penetrate cells, unlike inhibitors of reverse transcriptase, integrase or protease. Thirdly, because viral entry consists of separate steps, there are multiple targets for entry inhibitors, which is a safeguard against cross-resistance [12].

**Figure 1:**
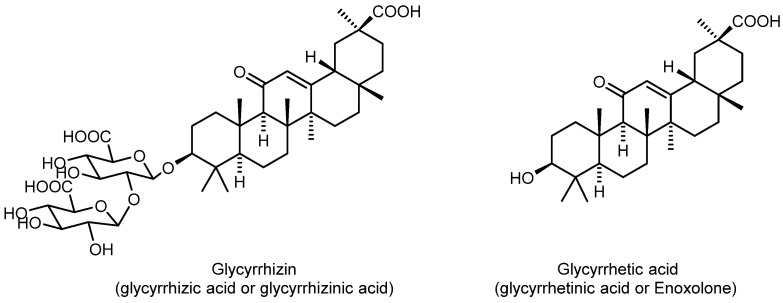
Structures of Glycyrrhizin and Glycyrrhetic acid.

There have been many studies [13,14] on potential new antiviral agents based on compounds of natural origin, in particular targeting the SARS-CoV-2 virus [15,16]. Natural compounds and their derivatives may be a valuable source of inhibitors of both HIV and coronaviruses [17]. Among these, Glycyrrhizin (glycyrrhizic acid or glycyrrhizinic acid, Glyc) (Figure 1) is a particularly interesting candidate as a potential broad-spectrum antiviral agent. Glycyrrhizin is the main triterpene glycoside found in the roots of licorice (*Glycyrrhizaglabra* L.) and Ural (*G. uralensis* Fisher). The corresponding aglycone, Glycyrrhetic acid (glycyrrhetinic acid or Enoxolone, GA), is a pentacyclic triterpenoid derivative of the beta-amyrin type, obtained by hydrolysis of glycyrrhizin.

Reports describing the antiviral activity of Glycyrrhizin against HIV-1 isolates were published already in the 1990s [18]. Furthering the understanding of this compound, Sasaki et al. later showed that glycyrrhizic acid does not affect the virus directly but inhibits HIV-1 replication in peripheral blood mononuclear cell (PBMC) culture by inducing the production of β-chemokines, which bind to the CCR5 chemokine receptor, thereby preventing the virus from entering PBMC [19].

The activity of Glyc and GA against SARS-CoV-2 has been investigated by several research groups [20,21,22,23]. However, there is still no consensus on the mechanisms of action that Glyc exerts against this virus.

Derivatives of GAs with a potentially stronger antiviral effect than the parent molecules have also been studied. Specifically, the amide of glycyrrhizic acid with 5-aminouracil [24], di- and/or trinicotinate of glycyrrhizic acid and their salts [25,26] have all been investigated for their anti-HIV activity. In contrast, glycyrrhizic acid nicotinates have not been investigated, neither in terms of their exact structures nor of their mechanisms of action.

In this report, we present the results of our preparation of nicotinates glycyrrhizic and glycyrrhetic acids and of our experiments on their antiviral activity against both HIV-1 (using the env-pseudovirus system) and SARS-CoV-2.

## 2. Results and Discussion

### 2.1. Chemistry

**Figure 2:**
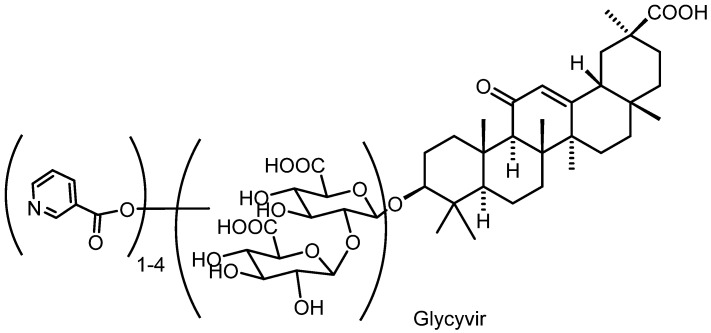
General formula of Glycyvir.

Glycyrrhizinic acid is isolated from natural sources that contain related saponins in addition to its main component, Glyc itself. The product we obtained in our synthesis is a multi-compound mixture of Glyc-based derivatives containing Glyc nicotinates, mainly mono-, di-, tri- and tetranicotinates. This mixture—which we named Glycivir—is depicted in general terms in Figure 2. Modern analytical methods were then employed to analyze this mixture in an attempt to harness the complexity of such analyses.

Our method of synthesis of Glycivir is a classical acylation of alcohol groups with nicotinic acid chlorohydride generated in situ. It offers several advantages; for instance, the process takes place in a single reaction vessel, without the addition or removal of any intermediates and without the use of any protective groups. As a result, the atomic efficiency of the synthesis is increased and there is no need to check for the presence of residual protecting groups in the final product. As demonstrated previously (RF Patent 2363703, 2009; RF Patent 2304145, 2007; RF Patent 2376312, 2009), this method of synthesis approach is very efficient.

The Glycyvir synthesis method described in the article is the result of optimizations to achieve high antiviral activity and low toxicity. After determining the optimal nicotinic acid/glycyrrhizic acid ratio, figuring out the optimal order and rate of mixing of the reagents, the residence time of the reaction mixture at various stages of the synthesis, and the acceptable temperature intervals at each process step, we obtained a well-reproducible process from synthesis to synthesis. It should be noted that the quality of the starting glycyrrhizic acid is important, since the suppliers do not particularly carefully analyze exactly what glycosylation of glycyrrhetic acid is in the preparation they supply. Usually, commercial preparations are mixtures of glycyrrhetic acid glycosides, and it is necessary to choose a supplier of exactly glycyrrhizic acid and not mixtures of unspecified compositions. However, this fact does not refer to the synthesis itself, but to the verification of the starting material.

Our method of synthesis of glycyrrhizic acid nicotinates does not allow for the selective production of individual compounds. This may be seen as a shortcoming. However, it may also prove to be an advantage; a “combination therapy” with different, but related nicotinates may well prove more effective in a broad-spectrum fight against different viruses, a “moving target” par excellence. Specifically, small changes in the structures of the synthesized compounds, described later in this report, besides having low toxicity, may well hinder the emergence of resistant variants. Indeed, unless one considers long-term evolutionary transformations, it seems extremely unlikely that the mechanisms of viral cell entry could be sufficiently modified through mutations to evade the inhibitory effects of different compounds that all have a common mechanism of action. In other words, the therapeutic rationale of Glycivir relies on the presence of small but significant structural variants that would collectively prevent the replication of whatever viral variants may arise through mutations. As a corollary, the spread of mutant viral variants would also be prevented.

**Figure 3:**
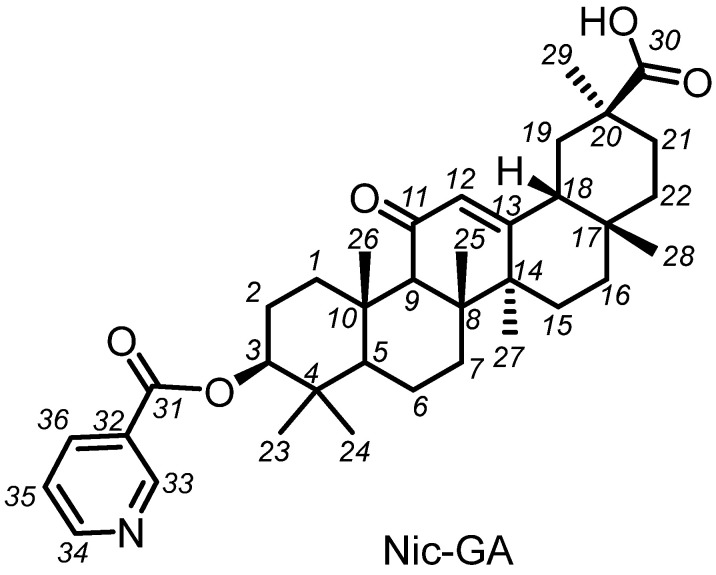
Structure of nicotinamide of glycyrrhetic acid.

In order to study the biological activities of the glycosidic fragment, we synthesized Nic-GA, a nicotinamide of glycyrrhetic acid. The structure of the individual compound is shown in Figure 3.

### 2.2. Determination of Qualitative Composition of Glycyrrhizic Acid Derivatives Using LC-MS/MS

#### Development of a Method for the Screening of Glycyrrhyzinic Acid Derivatives Using LC-MS/MS

**Figure 4:**
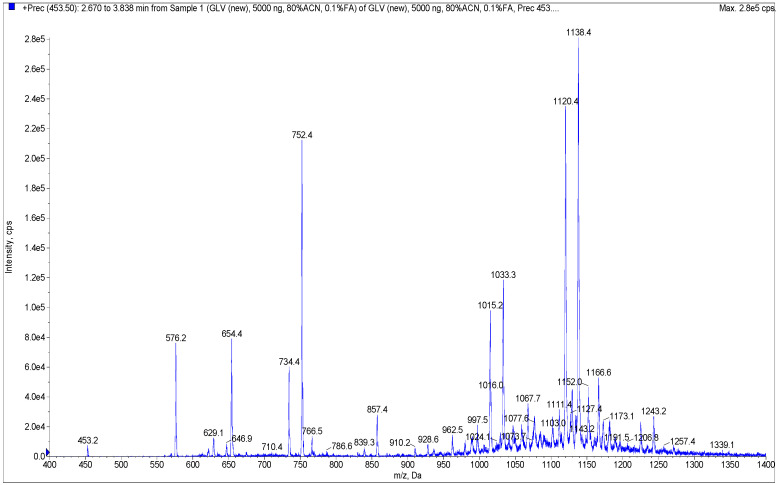
Mass spectrum of a sample obtained after the reaction of glycyrrhizinic acid and nicotinic acid.

In order to develop a method for the analysis of the products obtained in the reaction of glycyrrhizinic acid nicotination, several samples were analyzed using direct flow injection analysis. Weighings of samples (1–1.5 mg) were initially dissolved in a mixture of DMSO–EtOH (1:9) to obtain solutions of the same concentration (1 mg/mL). Samples for testing at a concentration of 5 mcg/mL were prepared through serial dilutions in a mixture of MeOH–H_2_O (8:2) containing 0.1% HCOOH. MS analyses of these samples in Q1 mode (without fragmentation) using positive electrospray ionization revealed the presence of a number of compounds having molecular ions of *m*/*z* ca. 1000 Da and higher (Figure 4 and Figure S1, Supplementary Materials). These compounds comprise mono- and oligonicotinates of glycyrrhizinic acid and their dehydrated derivatives.

Collision-induced dissociation (CID) of some of the observed molecular ions yielded mass spectra with a characteristic fragment ion with *m*/*z* = 453.5 Da. This fragment, corresponding to glycyrrhetic acid, was also observed during the fragmentation of glycyrrhizinic acid [27,28]. The observed fragmentation pattern served as the basis for our subsequent strategy: the screening for derivatives of glycyrrhizinc acid of any nature using the “Precursor ion” mode. This strategy made it possible to find all molecules generating a specific ion of known *m*/*z*.

The mass spectrum shown in Figure 4 was recorded for one of the synthesized samples in the 400 to 1400 Da mass range. The sample contained a number of molecular ions, of varying intensities, forming the fragment ion of *m*/*z* = 453.5 Da. It should be noted that the intensity of the signals in the mass spectrum does not reflect the content of any compound in the sample. This mass spectrum was analyzed manually, generating a collection of 36 molecular ions of high and low (“questionable”) intensities with each forming the *m*/*z* = 453.5 Da fragment.

The next step in the development of our method involved multiple reaction monitoring (MRM) detection with the use of a chromatographic separation of the sample. All MRM transitions were based on the principle of “M + H^+^ → 453.5”, i.e., the transitions of all found molecular ions into a specific glycyrrhetinium fragment (all *m*/*z*’s of the molecular ions are listed in Table 1). All samples tested in vitro were analyzed accordingly. Chromatographic conditions were developed and optimized based on several preliminary runs.

**Figure 5:**
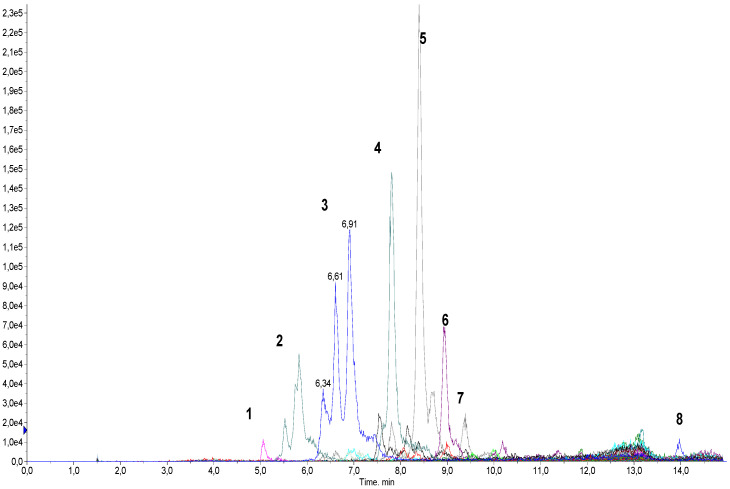
Chromatogram of Glycyvir recorded in MRM mode detecting 36 transitions (M + H^+^ of corresponding molecular ions are given in Table 1).

Figure 5 shows a chromatogram of a Glycyvir sample with colored lines representing different MRM transitions. It can be seen that one transition can form more than one peak in the chromatogram, often poorly separated from each other, if at all. We believe the close peaks correspond to isomeric mono- and oligonicotinates which are eluted together, forming a complicated chromatographic pattern.

To determine the relative content of each component in samples, peaks of each MRM transitions were integrated manually, and the integrated value was normalized relative to the total. The relative amount of each component is shown in Table 1. This approach does not take into account the presence of any impurities of non-glycyrrhizic nature. Nevertheless, we believe that the data obtained adequately reflect the composition of the samples under investigation.

The data we obtained show that nicotinate of glycyrrhettic acid is the major component of the Nic-GA sample. No other derivatives of the triterpenoid were detected.

One of the major components of Glycyvir is a mononicotinate of glycyrrhetic acid monoglucuronide; its content calculated on the total MRM ion current was about 26%. Taken together with glycyrrhizinic acid dinicotinate (ca. 24%), the two components constitute half of the entire sample. At the same time, glycyrrhizic acid trinicotinate comprised some 15%. The other compounds are likely products of simultaneous reactions of nicotination and intramolecular cyclisation resulting in the formation of a lactone group either in one or both glucuronide moieties.

As all analyses were carried out using a triple quadrupole system, it was impossible to establish brutto formulae of the components of the samples. Nevertheless, we suppose that the obtained results are quite reliable in terms of the relative content of the components. An investigation of the glycyrrhizic acid nicotinates obtained in this study as well as in other reactions will be the objective of our future studies.

### 2.3. Biological Activity

#### 2.3.1. Cytotoxicity

The cytotoxicity of the obtained compounds was assessed in TZM-bl cells using the MTT test. The preparations were diluted with DMEM medium so that their starting concentration was 1000 μM and the final volume of DMSO was no more than 1%. Additionally, we compared the cytotoxicity of DMEM and medium with 1% DMSO in TZM-bl cells and found no significant differences in cell survival. The measured CC50 values ranged from 7.8 to over 1000 μM (Table 2). Glycyrrhizic acid and GLC exhibited negligible toxicity against the TZM-bl cell line. In contrast, Nic-GA (glycyrrhetic acid nicotinamide) did exhibit some cytotoxic activity against the TZM-bl cell line. Since DMEM medium with 1% DMSO were not toxic to the cells, we did not include this control in the neutralization and TOA assays. A range of non-toxic concentrations was determined for each compound and used in subsequent experiments on their antiviral activity.

#### 2.3.2. Antiviral Activity against Infection by HIV-1 Pseudotyped Viruses

To evaluate the inhibitory activities of the synthesized compounds, HIV-1 pseudotyped viruses SF162.LS and OH0692 subtype B (which are included in the international reference panel), and pseudotyped viruses of the recombinant form CRF63_02A—16RU28 HIV-1 were used. Genetic variant CRF63_02A is currently the dominant type in Siberia and is rapidly spreading in the rest of the Russian Federation [28,29,30].

**Figure 6:**
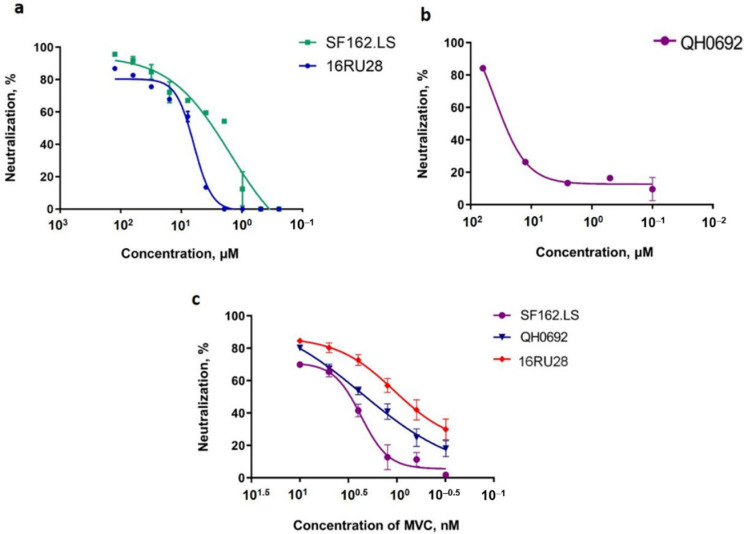
Neutralization of HIV-1 pseudoviruses in TZMbl-cells by Glycyvir and Maraviroc (MVC): the neutralization curves of Glycivir (**a**,**b**) and of Maraviroc (**c**). Maraviroc (MVC) was used as a positive control. The results presented were obtained from two independent experiments. Data are mean ± SD.

Based on the neutralizing assays we conducted, only Glycyvir exhibited inhibitory activity against HIV-1 pseudotyped viruses (Table 2). Glycyvir inhibited the entry into the target cells of both the pseudoviruses of subtype B and of the recombinant form of CRF63_02A (Figure 6).

Importantly, glycyrrhizic acid did not exhibit any inhibitory activity against the pseudoviruses SF162.LS, QH0692, or 16RU28. This finding confirms the results obtained by Sasaki et al. [19], which showed that glycyrrhizic acid does not directly affect the virus, but instead inhibits the replication of HIV-1 in PBMC cell cultures by inducing the production of β-chemokines that bind to the CCR5 chemokine receptor and thereby prevent the virus from entering PBMC cells.

The lack of inhibition by Nic-GA (nicotinamide-glycyrrhetic acid) suggests that the presence of the modified sugar portion may be important for antiviral activities or inhibitory properties.

#### 2.3.3. Time Dependency of the Inhibitory Effects of Glycivir

Time of addition assays (TOAs) were conducted to determine the stage at which Glycyvir interferes with HIV pseudoviral infection.

**Figure 7:**
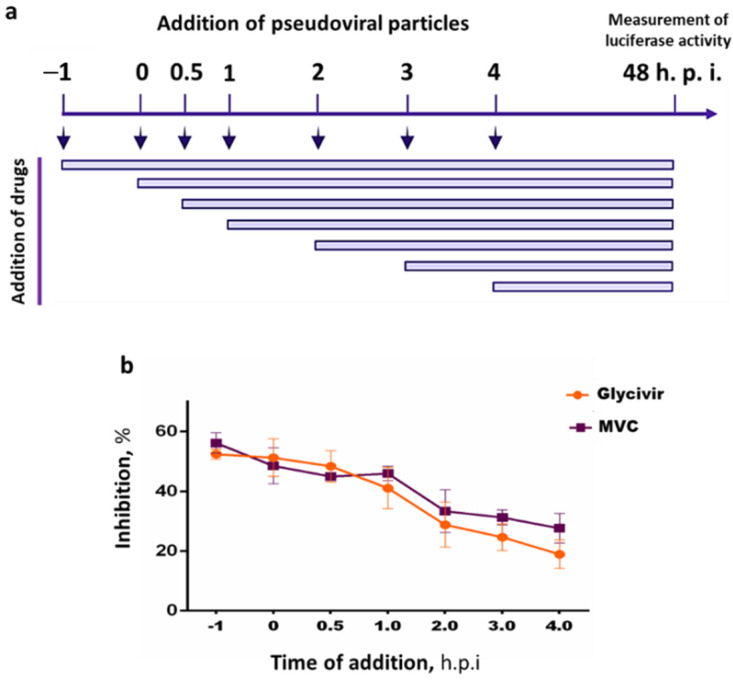
Time of addition analysis of the antiviral effects of Glycivir. (**a**) Timeline in the time of addition experiments. Glycyvir (3.9 µM) or Maraviroc (control drug) (5 nM) was added to TZMbl cells at t−1 h (1 h pre-infection), 0 h, 0.5 h, 1 h, 2 h, 3 h or 4 h (post-infection) to determine the inhibitory effects at different time points of administration. Antiviral activity was measured after 48 h by monitoring the luciferase activity. (**b**) Percentage inhibition caused by Glycivir or Maraviroc administrations at different time points. Error bars represent standard errors from two independent experiments.

As shown in Figure 7, Glycyvir exerted an inhibitory effect within the time interval of viral penetration, correlating with the established inhibition profile of MVC. This finding indicates that Glycyvir affects the stage at which the virus enters into the host cell. However, the target and the mechanism of inhibition remain unknown, warranting further research.

### 2.4. Antiviral Activity against SARS-CoV-2 Viruses

We have previously synthesized a number of novel compounds with a wide range of antiviral activities and that were all derived from natural substances [13]. Specifically, a wide range of antiviral activities were demonstrated in camphene derivatives [29], and high activities against influenza virus were measured in caryophyllene derivatives, prompting studies of their mechanism of action [30]. In line with this previous work, we decided to investigate to what extent Glycovir may inhibit SARS-CoV-2 viruses. That Glycivir may be an effective anti-SARS-CoV-2 agent is supported by the fact that the surface glycoproteins of coronaviruses and HIV are of the same type. Thus, they may well participate in identical or similar mechanisms important for viral entry and both be inhibited similarly.

Table 3 shows the results of inhibition experiments on three different SARS-CoV-2 viral strains [31].

The first SARS-CoV-2 strain, used as a prototype version in our experiments, is hCoV-19/Australia/VIC01/2020 lineages B. Viruses of this genetic lineage circulated during the first wave of the pandemic [32]. The second SARS-CoV-2 strain is hCoV-19/Russia/MOS-2512/2020, SARS-CoV-2 lineage B.1.1.7. This alpha variant was formally identified as a variant of concern by the UK government body Public Health England in December 2020, and named Variant of Concern 202012/01, although it came to be referred to as the Kent variant before the World Health Organization renamed it as the alpha variant in May 2021. This genetic variant has been spreading since late 2020. B.1.1.7 exhibits higher transmissibility, targeting a larger proportion of young people under 20 years of age [33].The third SARS-CoV-2 strain is hCoV-19/Russia/PSK-2804/2021, SARS-CoV-2 lineage B.1.617.2 (Delta). The SARS-CoV-2 delta (B.1.617.2) variant was first detected in India in October 2020. It has since rapidly become the predominant lineage, owing to its much higher transmissibility. The Delta variant has been associated with an increase in the severity of illness, as illustrated by a higher rate of hospitalization among patients with the Delta COVID-19 variant when compared to the Alpha variant, especially among the unvaccinated [34].

As shown in Table 3, unmodified glycyrrhizin exhibited no cytotoxicity and no antiviral activity against the three coronavirus strains. In contrast, Glicyvir proved highly active against the three strains, at levels comparable to that of Remdesivir, the reference drug. Glycivir’s anti-viral activity was highest against the Alpha and Delta strains. Nic-GA (Nicotinamide of glycyrrhetic acid), which proved less toxic to Vero cells than to Tzm-bl cells, did not exhibit any antiviral activity against the tested coronaviruses.

## 3. Materials and Methods

### 3.1. Synthesis of Glycivir Compounds

#### 3.1.1. NMR Spectral Analyses

^1^H and ^13^C NMR spectra were obtained in CDCl_3_ using a Bruker AM-400 spectrometer (operating frequencies of 400.13 MHz for ^1^H and 100.61 MHz for ^13^C) and a Bruker DRX-500 spectrometer (operating frequencies of 500.13 MHz and 125.76 MHz, respectively). The signals of the solvent (δH 7.24 and δC 76.9 ppm) were used as an internal standard. The structure of the obtained compounds was established on the basis of an analysis of ^1^H NMR spectra with the use of ^1^H-^1^H double resonance spectra and two-dimensional homonuclear ^1^H-^1^H correlation spectra, as well as an analysis of ^13^C NMR spectra recorded in the J-modulation (JMOD) mode, with non-resonant suppression of protons and two-dimensional spectra of heteronuclear ^13^C-^1^H correlation on direct and long-range spin-spin coupling constants (C-H COSY, ^1^J_C,H_ 135 Hz and COLOC, ^2,3^J_C,H_ 10 Hz, respectively).

#### 3.1.2. Synthesis of Glycyvir

A 100 mL double neck flask equipped with an efficient magnetic stirrer was placed in a glycerol bath, equipped with a thermometer. Pyridine (35 mL) was poured into the flask, followed by 5 g of nicotinic acid and the reaction mixture was heated to 40–45 °C (bath temperature), under constant stirring. Phosphorus pentachloride (2.8 g) was then quickly added to the resulting suspension still under stirring (to reduce watering). The mixture was than heated to 60 ± 3 °C and stirred at this temperature for 2 h (after about 30–40 min, the color of the reaction mixture changed from light to dark brown). The flask with the reaction mixture was then placed in an ice bath and the reaction mixture stirred until it cooled down to ~10–15 °C. Glycyrrhizic acid (1.5 g) was then added under stirring. The flask with the reaction mixture was placed in a bath with glycerol and heated to 55 ± 5 °C, under constant stirring, and kept at this temperature for 3 h. As a significant amount of precipitate was formed upon cooling, a strong magnetic stirrer and magnet were employed. When heated, a significant part of the precipitate dissolved. At the end of the reaction, the flask was removed from the bath and allowed to cool to ~30–40 °C. The suspension was then poured into a 150 mL glass and ~15 mL of crushed ice prepared from distilled water was added; distilled water was used to transfer the sediment remaining on the walls of the flask. The contents of the glass were mixed, and concentrated hydrochloric acid diluted with an equal volume of cold water (~60 mL of dilute acid per beaker) was added until a weakly acidic pH (~6) was reached. The mixture was left overnight (~12 h) for complete precipitation and the light brown precipitate was filtered out. This precipitate was repeatedly washed with distilled water (5 × 15 mL) and thoroughly dried on the filter. During the drying process of the sludge, large conglomerates were formed, which had to be crushed to accelerate drying. The dried sludge (~1.8 g) was dissolved in a mixture of 11 mL of chloroform and 2.2 mL of methanol (chloroform–methanol = 5:1 *v*/*v*). The dissolution process was slow, requiring 1–2 h under vigorous stirring. The dissolved solution was filtered, and the remaining precipitate discarded, after an additional wash with 1 mL of a chloroform and methanol mixture (as above). Diethyl ether (12 mL) was added to the filtered solution, causing intense precipitation. The mixture was stirred and left for 1 h for the complete formation of the precipitate. The formed precipitate was then filtered off, washed with 1.5 mL of diethyl ether and dried on a filter. Product yield was 1.630 g. Elemental analysis: C, 61.42; H, 6.09; N, 3.96. Nicotinic acid calculated Elemental Analysis: C, 58.54; H, 4.09; N, 11.38. Mononicotinate calculated Elemental Analysis: C, 62.12; H, 7.06; N, 1.51. Dinicotinate calculated Elemental Analysis: C, 62.78; H, 6.63; N, 2.71. Trinicotinate calculated Elemental Analysis: C, 63.31; H, 6.29; N, 3.69. Tetranicotinate calculated Elemental Analysis: C, 63.76; H, 6.00; N, 4.51. NMR ^1^H δ, p.p.m.: 0.81, 1.11, 1.17 and 1.37 methyl groups in the glycyrrhetic acid residue, broad signals at 7.5, 8.4, 8.7 and 9.1 protons in the aromatic nucleus of nicotinic acid. NMR ^13^C δ, p.p.m.: 16.03, 18.47, 23.17, 28.05 methyl groups in the glycyrrhetic acid residue, 163.95, 127.01, 151.93, 149.83, 123.70, 137.30 nicotinic acid residue.

#### 3.1.3. Synthesis of Nic-GA

A 100 mL double neck flask equipped with an efficient magnetic stirrer was placed in a glycerol bath, equipped with a thermometer. Pyridine (35 mL) was poured into the flask, followed by 5 g of nicotinic acid and the reaction mixture was heated to 40–45 °C (bath temperature), under constant stirring. Phosphorus pentachloride (2.7 g) was then quickly added to the resulting suspension still under stirring (to reduce watering). The mixture was than heated to 60 ± 3 °C and stirred at this temperature for 2 h (after about 30–40 min, the color of the reaction mixture changed from light to dark brown). The flask with the reaction mixture was then placed in an ice bath and the reaction mixture stirred until it cooled down to ~10–15 °C. Glycyrrhetic acid (GA, 1.5 g) was then added under stirring. The flask with the reaction mixture was placed in a bath with glycerin and heated to 55 ± 5 °C, under constant stirring, and kept at this temperature for 3 h. The further synthesis was carried out as for Glycivir above—up to, but not including the dissolution in a chloroform–methanol mixture.

The dried slurry weighed 1.335 g. Based on HPLC data, the precipitate contained an additional substance, which is probably a mixed anhydride of the target product with nicotinic acid. This precipitate was resuspended in 50 mL of water, and 10 mL of glacial acetic acid was added and vigorously stirred at 60 °C for 6 h. The mixture was evaporated to dryness and washed on a filter with water to remove residual nicotinic acid. Product weight was 1.010 g, and the yield 55%. Calculated Elemental Analysis: C, 75.10; H, 8.58; N, 2.43. Found Elemental Analysis: C, 74.65; H, 8.63; N, 2.45. Found, *m*/*z*: 575.3609 [M]^+^. C_36_H_49_NO_5_. Calculated M 575.3611. NMR ^1^H δ, p.p.m. (J, Hz): 0.81 s (C^28^H_3_), 0.84 d.d (H^5a^, J_5a,6a_ 12.5, J_5a,6a_ 1.5), 0.93 s (C^23^H_3_,), 1.01 s (C^24^H_3_), 1.01 d.m (H^15e^, J_15e,15a_ 13.8), 1.10 d.d.d (H^1a^, J_1a,1e_ 13.5, J_1a,2a_ 13.5, J_1a,2e_ 3.7), 1.12 s (C^26^H_3_), 1.18 s (C^29^H_3_), 1.20 s (C^25^H_3_), 1.21 d.m (H^16e^, J_16e,16a_ 13.8), 1.27–1.39 m (H^7^, H^21a^, 2H^22^), 1.37 s (C^27^H_3_), 1.42 d.d.d.d (H^6a^, J_6a,6e_ 13.5, J_6a,7a_ 13.5, J_6a,5a_ 12.0, J_6a,7e_ 3.2), 1.54–1.71 m (H^6e^, H^2e^, H^7′^), 1.77 d.d (H^19a^, J_19a,19e_ 13.5, J_19a,l8a_ 13.5), 1.81 d.d.d.d (H^2a^, J_2a,2e_ 13.5, J_2a,1a_ 13.5, J_2a,3a_ 11.7, J_2a,1e_ 3.7 Гц), 1.84 d.d.d (H^16a^, J_16a,16e_, 13.8, J_l6a,15a_ 13.8, J_16a,15e_ 4.5), 1.92 d.d.d (H^19e^, J_19e,19a_ 13.5, J_19e,l8a_ 4.2, J_19e,21e_ 2.7 Гц), 1.97 d.m (H^21e^, J_21e,21a_ 10 Гц), 2.00 d.d.d (H^15a^, J_15a,15e_ 13.8, J_15a,16a_ 13.8, J_15a,16e_ 4.8 Гц), 2.19 d.d (H^18a^, J_18a,19a_ 13.5, J_18a,19e_ 4.2), 2.38 c (H^9a^), 2.84 d.d.d (H^1e^, J_1e,1a_ 13.5, J_1e,2a_ 3.7, J_1e,2e_ 3.0 Гц), 4.77 д.д (H^3a^, J_3a,2a_ 11.7, J_3a,2e_ 4.7), 5.71 s (H^12^), 7.40 m (H^35^), 8.30 m (H^36^), 8.78 m (H^34^), 9.21 s (H^33^). NMR ^13^C δ, p.p.m.: 38.61 (C^1^), 23.48 (C^2^), 82.00 (C^3^), 38.28 (C^4^), 54.94 (C^5^), 17.26 (C^6^), 32.55 (C^7^), 43.67 (C^8^), 61.53 (C^9^), 36.83 (C^10^), 200.08 (C^11^), 128.29 (C^12^), 169.53 (C^13^), 45.32 (C^14^), 26.28 (C^15^), 26.36 (C^16^), 31.74 (C^17^), 48.12 (C^18^), 40.83 (C^19^), 43.10 (C^20^), 30.84 (C^21^), 37.61 (C^22^), 28.10 (C^23^), 16.84 (C^24^), 16.28 (C^25^), 18.56 (C^26^), 23.25 (C^27^), 28.41 (C^28^), 28.38 (C^29^), 181.01 (C^30^), 164.64 (C^31^), 126.85 (C^32^), 152.63 (C^33^), 150.26 (C^34^),123.39 (C^35^), 137.33 (C^36^).

#### 3.1.4. LC-MS/MS Analysis

HPLC-MS/MS analyses were performed using a Shimadzu LC-20AD Prominence chromatograph equipped with a SIL-20AC cooled autosampler and a gradient pump. A column filled with a reversed-phase ProntoSil 120-AQ C18 sorbent (Econova, Novosibirsk, Russia) was used. Water with 0.1% HCOOH was used as the mobile phase A, and ACN containing 0.1% HCOOH was used as the mobile phase B. The gradient was as follows: 0 min—5% B; 1 min—5% B; 2 min—95% B; 5 min—100% B; the flow rate was 300 μL/min; the injection volume was 10 μL. The column was then equilibrated for the next analysis.

Mass spectrometric detection was carried out on a 6500 QTRAP mass spectrometer (SCIEX, Framingham, MA, USA) using electrospray ionization. The following conditions were used: positive MRM mode, CUR (curtain gas) = 30 psi, CAD (collision-activated dissociation gas) = High, IS (ion source voltage) = 5500 V, TEM (temperature) = 350 °C, GS1 (sprayer gas) = 20 psi, GS2 (evaporator gas) = 20 psi, dwell time = 100 msec. The instrument was controlled, and information was collected using Analyst 1.6.3 software (AB SCIEX), chromatograms were processed using MultiQuant 2.1 software (AB SCIEX, Framingham, MA, USA).

### 3.2. Cell Lines and Viral Strains

TZM-bl cells were obtained from the National Institutes of Health AIDS Reagent Repository and cultured in DMEM (Gibco, Waltham, MA, USA) supplemented with 10% (*v*/*v*) fetal bovine serum (Gibco), 200 µg/mL L-glutamine and 50 µg/mL gentamicin. HEK-293T cells were obtained from the Culture Collection of SRC VB “Vector” Rospotrebnadzor and maintained in DMEM (Gibco) supplemented with 10% (*v*/*v*) fetal bovine serum, 200 µg/mL L-glutamine and 50 µg/mL gentamicin. The HIV Env clones pSF162.LS (ARP-10463) and pQH0692 (ARP-11018) and backbone vector pSG3∆env (ARP-11051) were obtained from the National Institutes of Health AIDS Reagent Repository. Plasmid p16RU28 encoding the surface glycoprotein gene ofHIV-1 CRF63_02A was constructed at SRC VB “Vector” Rospotrebnadzor. Studies using the HIV-1 pseudoviruses have been performed in laboratories with BSL-2 containment. Pseudoviruses were prepared by transfecting HEK293T cells with plasmids encoding env genes (pSF162.LS, pQH0692 or p16RU28) and an Env-deficient HIV-1 backbone vector (pSG3Δenv) using Lipofectamine 3000 reagent (Invitrogen). Pseudoviral particles were collected by filtering the culture medium through a 0.45-micron filter; aliquots of 1 mL were prepared and stored at −80 °C [31].

#### 3.2.1. MTT Cytotoxicity Assays

TZM-bl cells were seeded into 96-well culture plates (100 μL of cell suspension or 10^4^ p cells per well) and placed in a CO_2_ incubator. The test compounds were dissolved in DMSO at a concentration of 100 mM. After 24 h of incubation, various concentrations of the test compounds (1000, 500, 250, 125, 62.5, 31.25, and 15.6 μM) were added by titration to the TZM-bl cell culture, in triplicates. DMSO was added to the control wells at a concentration of no more than 1%. The plates with the introduced drugs were incubated in a CO_2_ incubator at 37 °C under 5% CO_2_. After 72 h of incubation, 20 μL of MTT working solution (5 mg/mL) was added to each well and the plates were again incubated under the same conditions. After 2 h, the plates were removed from the CO_2_ incubator, and the medium in each well was replaced with DMSO solution (50 μL per well). The plates were shaken gently to dissolve the formazan crystals. The optical density of each well was determined at 570 nm using a Varioscan LUX plate reader (Thermo scientific). The survival rate of TZM-bl cells in the presence of the tested compounds was calculated using the formula: (OD of experimental wells—OD of medium)/(OD of control wells—OD of medium) × 100%, where OD is optical density.

#### 3.2.2. Evaluation of the Inhibitory Activity of Drugs Using HIV-1 Pseudoviruses

The anti-HIV-1 activity of the tested compounds was performed using the TZM-bl assay, carried out in 96-well culture plates, as described previously [32]. Briefly, serial 2-fold dilutions of the tested compounds were prepared in DMEM growth medium, 200 TCID_50_ pseudovirus were added to each well and incubated for 1 h at 37 °C under 5% CO_2_. Trypsinized TZM-bl cell suspension was then added (100 μL of 2 × 10^5^ cells/mL) and plates were further incubated at 37 °C under 5% CO_2_. After 48 h, the luminescence signal was recorded using the luciferase assay system (Promega). The percent inhibition for each compound was calculated as the ratio between the relative luminescence unit (RLU) values of the test wells (sample + pseudovirus + cells) and the virus control (pseudovirus + cells). Samples were tested in triplicates, and the experiment was repeated twice.

#### 3.2.3. Time-of-Addition Assay

A time-of-addition study was conducted using a single-round replication assay. Briefly, TZM-bl cells were seeded in 96-well plates (2 × 10^4^ cells/well). After 24 h, SF162.LS pseudoviral particles were added to the cell monolayer. Maraviroc (MVC, Sigma-Aldrich, St. Louis, MO, USA) was used as a reference (control) drug. The tested compounds and control drug were then added to the cells at various points in time. Inhibition of pseudoviral infection was assessed after 48 h by measuring the level of luminescence, as above. Samples were tested in triplicates, and the experiment was repeated twice.

#### 3.2.4. Evaluation of the Antiviral Activities against SARS-CoV-2 Viruses

Vero E6 cells were used in the experiment (cells from the epithelium of the kidney of an African green monkey) (collection of SRC VB “Vector” Rospotrebnadzor, RF). The cells were cultured in a DMEM medium (Gibco) with L-glutamine, with 10% fetal calf serum (Gibco) and antibiotic-antimycotic (Gibco) at 37 °C, 5% CO_2_. Studies using the SARS-CoV-2 virus have been performed in laboratories with BSL-3 containment.

The study was carried out using three coronavirus strains: SARS-CoV-2 nCoV/Victoria/1/2020 (GISAID ID: EPI_ISL_406844, lineages B), hCoV-19/Russia/MOS-2512/2020, (GISAID ID: EPI_ISL_6565012, lineages B1.1.7), and hCoV-19/Russia/PSK-2804/2021 (GISAID ID: EPI_ISL_7338814, lineages B1.617.2) (State collection of pathogens of viral infections and rickettsioses of SRC VB “Vector” Rospotrebnadzor, RF). The viruses were grown in a Vero E6 cell culture. The infectivity titer of the virus stock was measured at 6.5 lg TCD_50_/mL. Vero E6 cells were grown in 96-well culture plates to a confluence of at least 95%. Samples were dissolved in dimethyl sulfoxide (DMSO) to a concentration of 10 mg/mL. Remdesivir was used as a control drug. Evaluation of the effective (IC_50_) concentrations of the compounds was carried out in the test to reduce the cytopathic effect on cells. Serial threefold dilutions of the compounds were prepared, starting at a concentration of 600 μg/mL. The experiment used coronavirus 2019-nCoV at a dose of 100 TCD_50_ per well.

Inhibitory activities and the toxicities of the tested compounds were assessed simultaneously. Specifically, dilutions of the compounds were added to the wells in the culture plates containing a monolayer of cells. Plain medium (to determine the toxic concentration of the tested compounds) or medium containing a virus (to determine inhibitory activities) were then added. The culture plates were incubated at 37 °C for 4 days, then stained using the MTT assay protocol. The results were recorded with Thermo Scientific Multiskan FC; data processing was carried out using the SOFTmax PRO 4.0 program using a 4-parameter analysis method. The 50% toxic concentration (CD_50_) and the 50% inhibition (IC_50_) concentrations were both determined. The selectivity index (SI), as the ratio of the toxicity of the compound and the inhibitory activity, was also calculated (Table 3).

### 3.3. Statistical Analysis

All statistical analyses were performed using GraphPad Prism software.

## 4. Conclusions

In this work, Glycyvir, a mixture of nicotinates of glycyrrhizic acid, was obtained, and characterized in terms of its composition and its anti-viral activities. Glycyvir proved effective against three different strains of SARS-CoV-2 virus and HIV-1 pseudoviruses, while exhibiting low toxicity. Time-dependence studies of the inhibitory activity of Glycyvir on HIV pseudoviruses suggest that inhibition takes place at the stage of viral entry into the host cell. These findings are prerequisites for the development of a low-toxicity, broad-spectrum anti-viral agent that would prevent the infection of cells targeted by viruses with common or similar mechanisms of viral entry. In addition, the fine heterogeneity of the compounds that make Glycivir may be a safeguard against the emergence of resistant mutant viral strains. Glycivir thus exhibits all the characteristics of a dual drug that would protect AIDS patients and people with HIV against SARS-CoV-2 while simultaneously acting as a therapeutic agent against their disease.

## Figures and Tables

**Table 1:** Molecular ions of the components as well as their relative content in samples (normalized to the total ion current recorded in MRM mode).

Component Name and/or Molecular Weight, Da ^a^	M + H^+^	Amount of the Component in Sample, % ^b^	
		Glycyvir	Nic-GA
glycyrrhetinic acid nicotinate (575.2, **8**)	576.2	0.69	**99.87**
653.4	654.4	0.04	0.02
Mononicotinate of glycyrrhetinic acid lactouronide (733.4)	734.4	0.49	0.01
Mononicotinate of glycyrrhetinic acid glucuronide (751.4, **5**)	752.4	**26.14 ^d^**	N.D. ^c^
765.5	766.5	N.D.	N.D.
Glycyrrhizinic acid (822.5, **1**)	823.5	0.79	N.D.
838.3	839.3	0.02	N.D.
856.5	857.5	0.34	N.D.
mono-nic, mono-lact (909.5)	910.5	**1.08**	N.D.
mono-nic (927.0, **2**)	928	**8.80**	N.D.
955.3	956.3	0.30	N.D.
961.5	962.5	0.15	N.D.
979.2	980.2	0.02	N.D.
di-nic, di-lact (996.5)	997.5	N.D.	N.D.
998	999	N.D.	N.D.
di-nic, mono-lact (1014.4)	1015.4	**5.21**	N.D.
di-nic (1032.4, **3**)	1033.4	**23.91**	N.D.
1060.5	1061.5	N.D.	N.D.
1067	1068	**1.81**	0.01
1075.9	1076.9	0.42	N.D.
1090	1091	0.90	0.01
tri-nic, di-lact (1101.2)	1102.2	**1.17**	0.01
1110.3	1111.3	**1.69**	0.01
tri-nic, mono-lact (1119.3, **6**)	1120.3	**6.10**	N.D.
1128.4	1129.4	**1.27**	0.01
tri-nic (1137.4, **4**)	1138.4	**15.48**	N.D.
1151	1152	0.12	0.03
1165.2	1166.2	**1.02**	N.D.
1171.6	1172.6	0.27	N.D.
1185.9	1186.9	0.58	N.D.
1189.1	1190.1	N.D.	N.D.
1194.6	1195.6	0.73	0.01
1223	1224	0.36	N.D.
tetra-nic, mono-lact (1224.3)	1225.3	N.D.	N.D.
tetra-nic (1242.6)	1243.6	0.09	N.D.
1341.9	1342.9	0.02	N.D.

^a^ The structures of the main compounds are given in the Supplementary Materials, Figures S1–S11; ^b^ relatively to the total area of all peaks; ^c^ N.D.—not determined; ^d^ content of the components above 1% is typed in boldface.

**Table 2:** Inhibitory activity of Glycivir and derivatives against HIV-1 env-pseudoviruses.

Agent	CC_50_	SF162.LS		OH0692		16RU28	
	µM	IC_50_. µM	SI	IC_50_. µM	SI	IC_50_. µM	SI
Glycyrrhizin	>1000	NA	-	NA	-	NA	-
Glycyvir	>1000	2.88 ± 0.12	347	27.5 ± 2.8	36	6.91 ± 0.23	138
Nic-GA	<7.8	NA	-	NA	-	NA	-
MVC	>1000	0.002	5 × 10^3^	0.002	5 × 10^3^	0.0016	6 × 10^3^

NA: no activity; MVC, Maraviroc, is confirmed HIV entry inhibitor. Each experiment was conducted three times and data were expressed as means ± SD.

**Table 3:** Antiviral activities of the Glycivir and derivatives against SARS-CoV-2 virus strains obtained on Vero E6 cell culture.

Agent	CC_50_	hCoV-19/Aust/VIC01/2020 ^a^		hCoV-19/Russ/MOS/2020 ^b^		hCoV-19/Russ/PSK/2021 ^c^	
	µM	IC_50_, µM	SI	IC_50_, µM	SI	IC_50_, µM	SI
Glycyrrhizin	>1000	NA	-	NA	-	NA	-
Glycyvir	334.5 ± 18.5	8.3 ± 1.10	40	2.2 ± 0.14	152	3.8 ± 0.41	88
Nic-GA	101.3	NA	-	NA	-	NA	-
Remdesivir	710.9 ± 21.2	3.3 ± 0.12	215	1.5 ± 0.14	473	1.9 ± 0.22	359

^a^ Virus strain—hCoV-19/Australia/VIC01/2020, GISAID ID: EPI_ISL_406844, genetic lineages by GISAID: B; ^b^ virus strain—hCoV-19/Russia/MOS-2512/2020, GISAID ID: EPI_ISL_6565012, genetic lineages by GISAID: B1.1.7; ^c^ virus strain—hCoV-19/Russia/PSK-2804/2021, GISAID ID: EPI_ISL_7338814, genetic lineages by GISAID: B1.617.2. NA—no activity.

## Data Availability

Not applicable.

## Supplementary Materials

The following are available online, Figures S1–S11: Glycivir—components of the mixture; Figures S12–S17: Primary data on antiviral activity against the virus SARS-CoV-2.

## Sample Availability

Samples of the compounds are not available from the authors.

## References

[1] https://www.who.int/news-room/fact-sheets/detail/hiv-aids.

[2] The Effects of the COVID-19 Pandemic on the HIV Response. https://www.unaids.org/en/resources/documents/2021/effects-of-covid19-pandemic-on-hiv-response.

[3] Cattaneo D., Cattaneo D., Gervasoni C., Corbellino M., Galli M., Riva A., Gervasoni C., Clementi E., Clementi E. (2020). Does lopinavir really inhibit SARS-CoV-2?. Pharmacol. Res..

[4] Patel T.K., Patel P.B., Barvaliya M., Saurabh M.K., Bhalla H.L., Khosla P.P. (2021). Efficacy and safety of lopinavir-ritonavir in COVID-19: A systematic review of randomized controlled trials. J. Infect. Public Health.

[5] Dang A., Vallish B., Dang S. (2020). Hydroxychloroquine and Remdesivir in COVID-19: A critical analysis of recent events. Indian J. Med. Ethics.

[6] Ayerdi O., Puerta T., Clavo P., Vera M., Ballesteros J., Fuentes M.E., Estrada V., Rodríguez C., Del Romero J., Del Romero J. (2020). Preventive Efficacy of Tenofovir/Emtricitabine Against Severe Acute Respiratory Syndrome Coronavirus 2 Among Pre-Exposure Prophylaxis Users. Open Forum Infect. Dis..

[7] Tompa D.R., Immanuel A., Srikanth S., Kadhirvel S. (2021). Trends and strategies to combat viral infections: A review on FDA approved antiviral drugs. Int. J. Biol. Macromol..

[8] Martinez M.A. (2021). Lack of Effectiveness of Repurposed Drugs for COVID-19 Treatment. Front. Immunol..

[9] Phanuphak N., Gulick R.M. (2020). HIV treatment and prevention 2019. Curr. Opin. HIV AIDS.

[10] Arts E.J., Hazuda D.J. (2012). HIV-1 Antiretroviral Drug Therapy. Cold Spring Harb. Perspect. Med..

[11] Lobritz M.A., Ratcliff A.N., Arts E.J. (2010). HIV-1 Entry, Inhibitors, and Resistance. Viruses.

[12] Xiao T., Cai Y., Chen B. (2021). HIV-1 Entry and Membrane Fusion Inhibitors. Viruses.

[13] Yarovaya O.I., Salakhutdinov N.F. (2021). Mono- and sesquiterpenes as a starting platform for the development of antiviral drugs. Russ. Chem. Rev..

[14] Musarra-Pizzo M., Pennisi R., Ben-Amor I., Mandalari G., Sciortino M.T. (2021). Antiviral Activity Exerted by Natural Products against Human Viruses. Viruses.

[15] Farooq S., Ngaini Z. (2021). Natural and Synthetic Drugs as Potential Treatment for Coronavirus Disease 2019 (COVID-2019). Chem. Afr..

[16] Diniz L.R.L., Perez-Castillo Y., Elshabrawy H.A., Filho C.d.S.M.B., de Sousa D.P. (2021). Bioactive Terpenes and Their Derivatives as Potential SARS-CoV-2 Proteases Inhibitors from Molecular Modeling Studies. Biomolecules.

[17] Anywar G., Akram M., Chishti M.A. (2021). African and Asian Medicinal Plants as a Repository for Prospective Antiviral Metabolites Against HIV-1 and SARS CoV-2: A Mini Review. Front. Pharmacol..

[18] Hirabayashi K., Iwata S., Matsumoto H., Mori T., Shibata S., Baba M., Ito M., Shigeta S., Nakashima H., Yamamoto N. (1991). Antiviral activities of glycyrrhizin and its modified compounds against human immunodeficiency virus type 1(HIV-1) and herpes simplex virus type 1(HSV-1) in vitro. Chem. Pharm. Bull..

[19] Sasaki H., Takei M., Kobayashi M., Pollard R.B., Suzuki F. (2002). Effect of Glycyrrhizin, an Active Component of Licorice Roots, on HIV Replication in Cultures of Peripheral Blood Mononuclear Cells from HIV-Seropositive Patients. Pathobiology.

[20] Li J., Xu D., Wang L., Zhang M., Zhang G., Li E., He S. (2021). Glycyrrhizic Acid Inhibits SARS-CoV-2 Infection by Blocking Spike Protein-Mediated Cell Attachment. Molecules.

[21] Yu S., Zhu Y., Xu J., Yao G., Zhang P., Wang M., Zhao Y., Lin G., Chen H., Chen L. (2021). Glycyrrhizic acid exerts inhibitory activity against the spike protein of SARS-CoV-2. Phytomedicine.

[22] van de Sand L., Bormann M., Alt M., Schipper L., Heilingloh C.S., Steinmann E., Todt D., Dittmer U., Elsner C., Witzke O. (2021). Glycyrrhizin Effectively Inhibits SARS-CoV-2 Replication by Inhibiting the Viral Main Protease. Viruses.

[23] Elebeedy D., Elkhatib W.F., Kandeil A., Ghanem A., Kutkat O., Alnajjar R., Saleh M.A., Abd El Maksoud A.I., Badawy I., Al-Karmalawy A.A. (2021). Anti-SARS-CoV-2 activities of tanshinone IIA, carnosic acid, rosmarinic acid, salvianolic acid, baicalein, and glycyrrhetinic acid between computational and in vitro insights. RSC Adv..

[24] Kondratenko R.M., Balina L.A., Mustafina S.R., Pljasunova O.A., Pokrovskij A.G., Tolstikov G.A. (2003). Glycyrrhizic Acid Amide with 5-Aminouracil Eliciting Anti-Hiv-Activity. Patent.

[25] Pokrovskij A., Salakhutdinov N., Tolstikov G. (2009). Method for Preparation of Pentanicotinate of Glycyrrhizic Acid Being Reproduction Inhibitor of Human Immunodeficiency Virus. Patent.

[26] Tolstikov G., Baltina L., Volcho K., Pljasunova O., Pokrovskij A., Salakhutdinov N. (2007). Di- and Trinicotinates of Glycyrrhizic Acid and Inhibitor of Human Immunodeficiency Virus Reproduction. Patent.

[27] Suzuki T., Tsukahara M., Akasaka Y., Inoue H. (2017). A highly sensitive LC-MS/MS method for simultaneous determination of glycyrrhizin and its active metabolite glycyrrhetinic acid: Application to a human pharmacokinetic study after oral administration. Biomed. Chromatogr..

[28] Ji B., Zhao Y., Yu P., Yang B., Zhou C., Yu Z. (2018). LC-ESI-MS/MS method for simultaneous determination of eleven bioactive compounds in rat plasma after oral administration of Ling-Gui-Zhu-Gan Decoction and its application to a pharmacokinetics study. Talanta.

[29] Sokolova A.S., Putilova V.P., Yarovaya O.I., Zybkina A.V., Mordvinova E.D., Zaykovskaya A.V., Shcherbakov D.N., Orshanskaya I.R., Sinegubova E.O., Esaulkova I.L. (2021). Synthesis and Antiviral Activity of Camphene Derivatives against Different Types of Viruses. Molecules.

[30] Volobueva A.S., Yarovaya O.I., Kireeva M.V., Borisevich S.S., Kovaleva K.S., Mainagashev I.Y., Gatilov Y.V., Ilyina M.G., Zarubaev V.V., Salakhutdinov N.F. (2021). Discovery of New Ginsenol-Like Compounds with High Antiviral Activity. Molecules.

[31] Tao K., Tzou P.L., Nouhin J., Gupta R.K., de Oliveira T., Kosakovsky Pond S.L., Fera D., Shafer R.W. (2021). The biological and clinical significance of emerging SARS-CoV-2 variants. Nat. Rev. Genet..

[32] Domingo P., de Benito N. (2021). Alpha variant SARS-CoV-2 infection: How it all starts. EBioMedicine.

[33] Volz E., Mishra S., Chand M., Barrett J.C., Johnson R., Geidelberg L., Hinsley W.R., Laydon D.J., Dabrera G., O’Toole Á. (2021). Assessing transmissibility of SARS-CoV-2 lineage B.1.1.7 in England. Nature.

[34] Twohig K.A., Nyberg T., Zaidi A., Thelwall S., Sinnathamby M.A., Aliabadi S., Seaman S.R., Harris R.J., Hope R., Lopez-Bernal J. (2021). Hospital admission and emergency care attendance risk for SARS-CoV-2 delta (B.1.617.2) compared with alpha (B.1.1.7) variants of concern: A cohort study. Lancet Infect. Dis..

