# Supplementary material for: Synthesis and In Vitro Study of Antiviral Activity of Glycyrrhizin Nicotinate Derivatives against HIV-1 Pseudoviruses and SARS-CoV-2 Viruses
Source: Molecules. 2022 Jan 4;27(1):295. doi: 10.3390/molecules27010295 (PMC8746574; doi:10.3390/molecules27010295)
Supplement: Supplementary file 1 [file molecules-27-00295-s001.zip › molecules-1540164-supplementary.pdf]

Synthesis and *in vitro* study of antiviral activity of glycyrrhizin nicotinate derivatives against HIV-1 pseudoviruses and SARS-CoV-2 viruses

Vladislav V. Fomenko[1‡], Nadezhda B. Rudometova[2‡], Olga I. Yarovaya[1,3*], Artem D. Rogachev[1,3], Anastasia A. Fando[3], Anna V. Zaykovskaya[2], Nina I. Komarova[1], Dmitriy N. Shcherbakov[2], Oleg V. Pyankov[2], Andrey G. Pokrovsky[3], Larisa I. Karpenko[2], Rinat A. Maksyutov[2] and Nariman F. Salakhutdinov[1,3]

1 Department of Medicinal Chemistry, N. N. Vorozhtsov Novosibirsk Institute of Organic Chemistry, Siberian Branch of the Russian Academy of Sciences, Lavrentiev Ave. 9, 630090 Novosibirsk, Russia

2 Department of Bioengineering and Department of Microorganisms Collection State Research Center of Virology and Biotechnology VECTOR, Rospotrebnadzor, 630559, Russia, Koltsovo, Novosibirsk Region

3 Zelman Institute for Medicine and Psychology, Novosibirsk State University, Pirogov Str., 1, Novosibirsk 630090, Russia

* Correspondence: ooo@nioch.nsc.ru

Supplementary materials

Glycivir - components of the mixture:

Glycyrrhizin (glycyrrhizic acid or glycyrrhizinic acid, Glyc), Fig. S1.

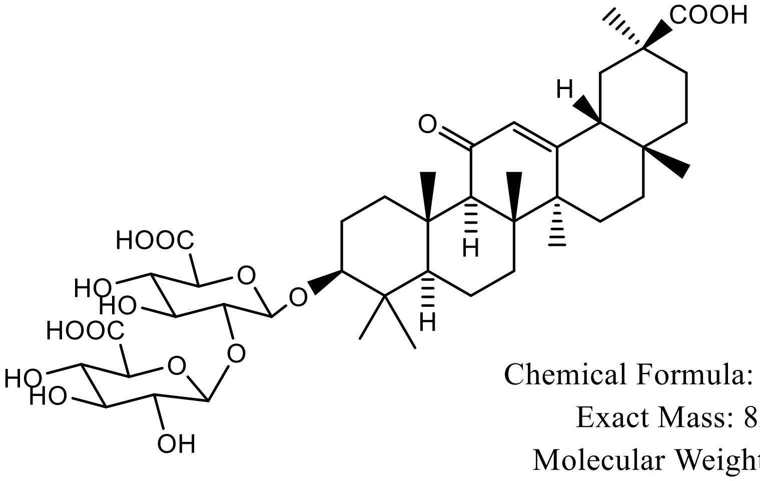

Chemical Formula: $C_{42}H_{62}O_{16}$
Exact Mass: 822,40
Molecular Weight: 822,94
m/z: 822.40 (100.0%), 823.41 (45.4%), 824.41 (10.1%), 824.41 (3.3%), 825.41 (1.5%)

Figure S1.

Mononicotinate of glycyrrhetinic acid glucuronide (the nicotinic fragment can be in any position of the sugar moiety), Fig. S2.

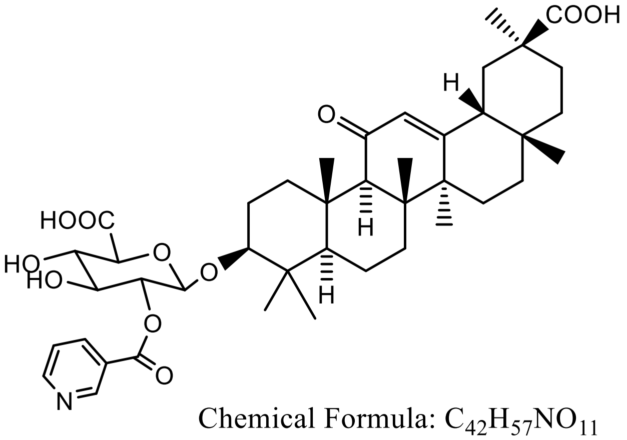

Chemical Formula: $C_{42}H_{57}NO_{11}$
Exact Mass: 751,39
Molecular Weight: 751,91
m/z: 751.39 (100.0%), 752.40 (45.4%), 753.40 (10.1%), 753.40 (2.3%), 754.40 (1.5%), 754.40 (1.0%)

Figure S2.

Mononicotinate of glycyrrhetinic acid lactouronide, Fig. S3.

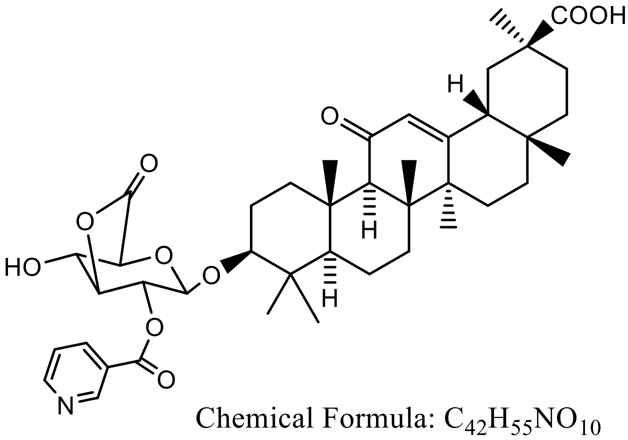

Chemical Formula: $C_{42}H_{55}NO_{10}$
Exact Mass: 733,38
Molecular Weight: 733,90
m/z: 733.38 (100.0%), 734.39 (45.4%), 735.39 (10.1%), 735.39 (2.1%)

Figure S3.

Mononicotinate of Glycyrrhizin (the nicotinic fragment can be in any position of the sugar moiety), Fig. S4.

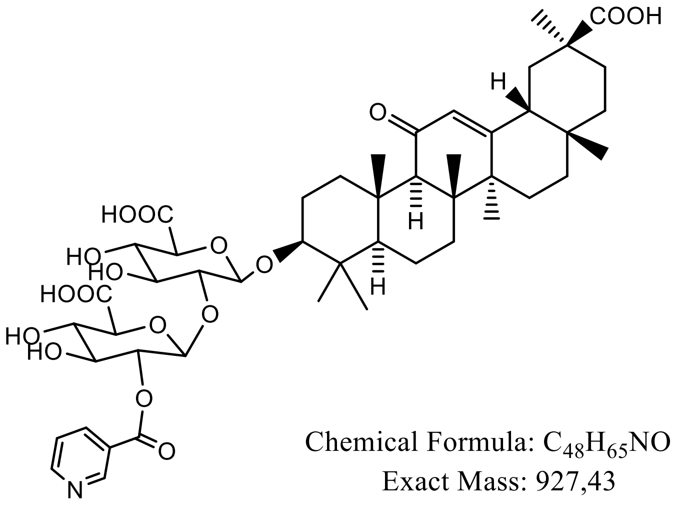

Chemical Formula: $C_{48}H_{65}NO_{17}$

Exact Mass: 927,43

Molecular Weight: 928,04

m/z: 927.43 (100.0%), 928.43 (51.9%), 929.43 (13.2%), 929.43 (3.5%), 930.43 (1.8%), 930.44 (1.4%)

Figure S4.

Mononicotinate, mono-lacton (the nicotinic fragment can be in any position of the sugar moiety), Fig. S5.

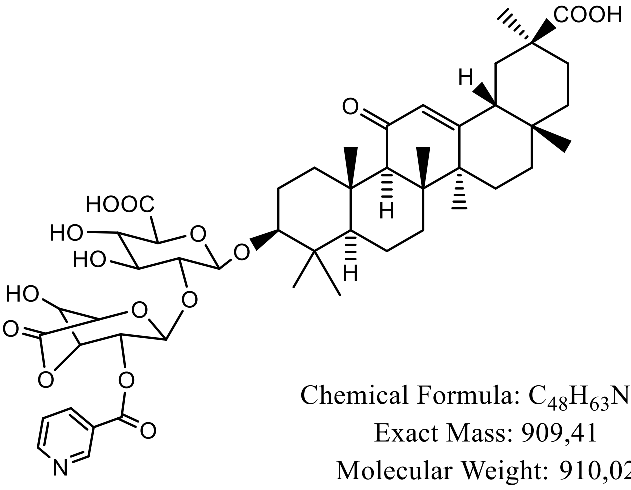

Chemical Formula: $C_{48}H_{63}NO_{16}$

Exact Mass: 909,41

Molecular Weight: 910,02

m/z: 909.41 (100.0%), 910.42 (51.9%), 911.42 (13.2%), 911.42 (3.3%), 912.42 (1.7%), 912.42 (1.4%)

Figure S5.

Dinicotinate of Glycyrrhizin (the nicotinic fragment can be in any position of the sugar moiety), Fig. S6.

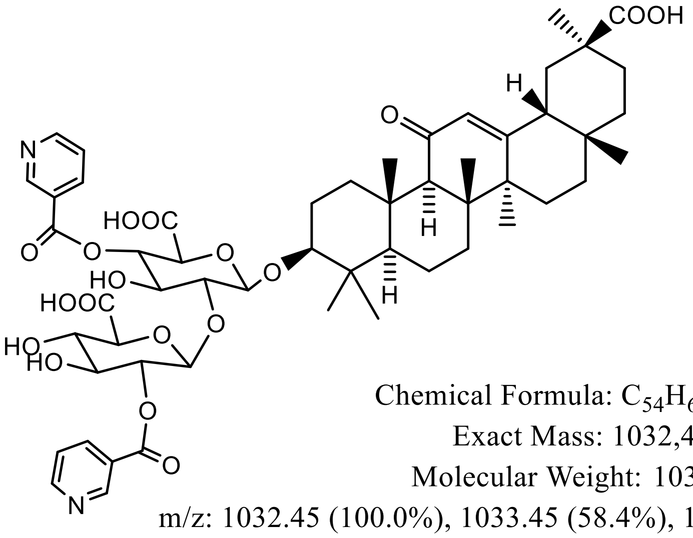

Chemical Formula: $C_{54}H_{68}N_2O_{18}$
Exact Mass: 1032,45
Molecular Weight: 1033,13
m/z: 1032.45 (100.0%), 1033.45 (58.4%), 1034.45 (16.7%), 1034.45 (3.7%), 1035.46 (2.3%), 1035.45 (2.2%)

Figure S6.

Dinicotinate, mono-lacton (the nicotinic fragment can be in any position of the sugar moiety), Fig. S7.

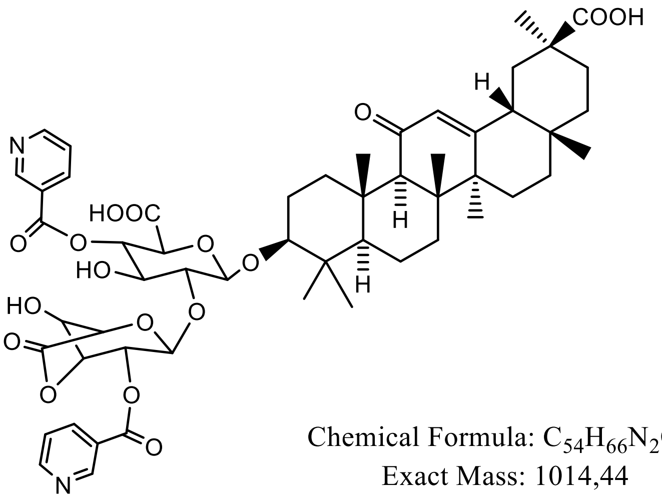

Chemical Formula: $C_{54}H_{66}N_2O_{17}$
Exact Mass: 1014,44
Molecular Weight: 1015,12
m/z: 1014.44 (100.0%), 1015.44 (58.4%), 1016.44 (16.7%), 1016.44 (3.5%), 1017.45 (2.3%), 1017.44 (2.0%)

Figure S7.

Trinicotinate, di-lacton (the nicotinic fragment can be in any position of the sugar moiety), Fig. S8.

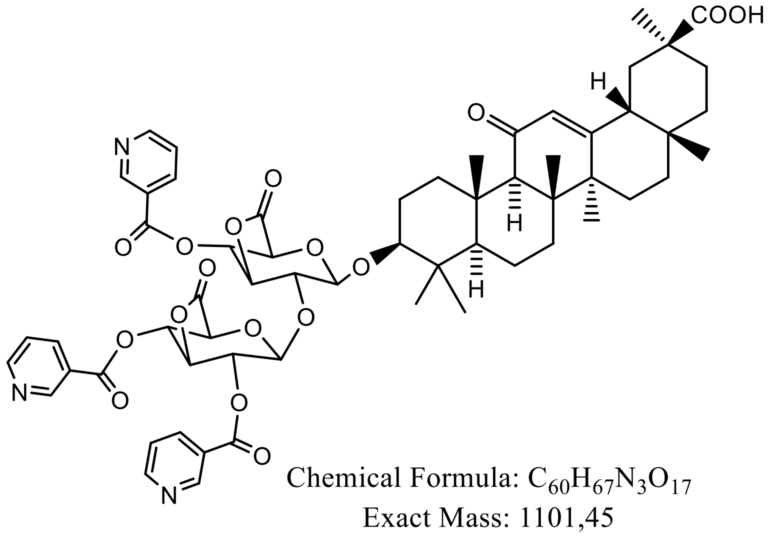

Chemical Formula: $C_{60}H_{67}N_3O_{17}$
Exact Mass: 1101,45
Molecular Weight: 1102,20
m/z: 1101.45 (100.0%), 1102.45 (64.9%), 1103.45 (20.7%), 1104.46 (4.3%), 1103.45 (3.5%), 1104.45 (2.3%), 1102.44 (1.1%)

Figure S8.

Trinicotinate, mono-lacton (the nicotinic fragment can be in any position of the sugar moiety), Fig. S9.

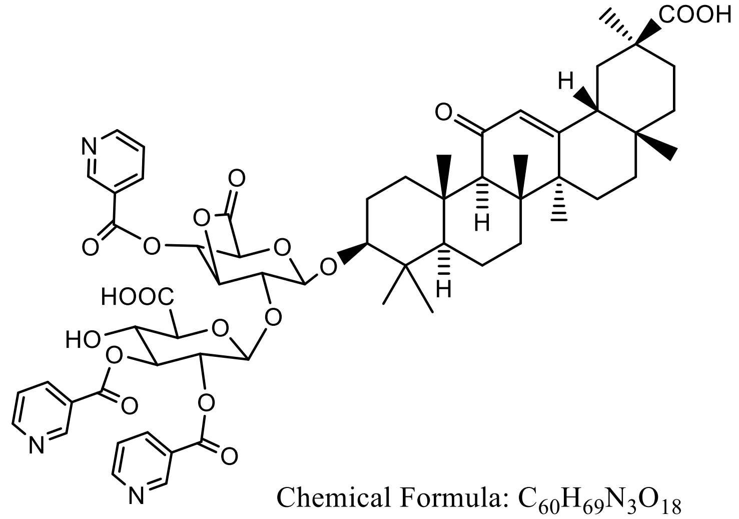

Chemical Formula: $C_{60}H_{69}N_3O_{18}$
Exact Mass: 1119,46
Molecular Weight: 1120,22
m/z: 1119.46 (100.0%), 1120.46 (64.9%), 1121.46 (20.7%), 1122.47 (4.3%), 1121.46 (2.5%), 1122.47 (2.4%), 1121.46 (1.2%), 1120.45 (1.1%)

Figure S9.

Trinicotinate of Glycyrrhizin, (the nicotinic fragment can be in any position of the sugar moiety), Fig. S10.

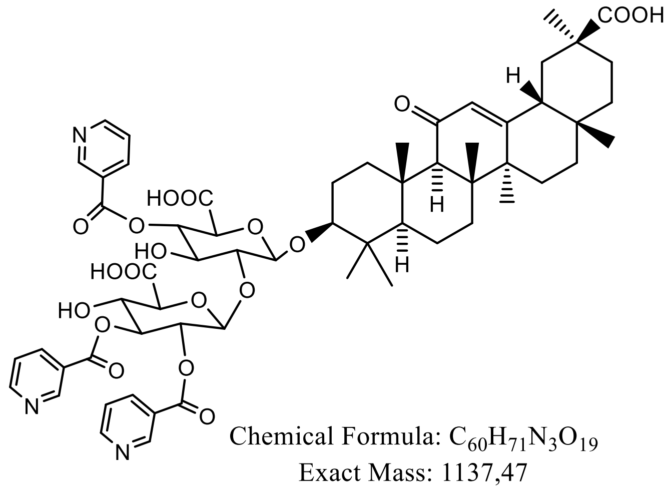

Chemical Formula: $C_{60}H_{71}N_{3}O_{19}$
Exact Mass: 1137,47
Molecular Weight: 1138,23
m/z: 1137.47 (100.0%), 1138.47 (64.9%), 1139.47 (20.7%), 1140.48 (4.3%), 1139.47 (3.9%), 1140.48 (2.5%), 1138.47 (1.1%)

Figure S10.

Tetranicotinate of Glycyrrhizin, (the nicotinic fragment can be in any position of the sugar moiety), Fig. S11.

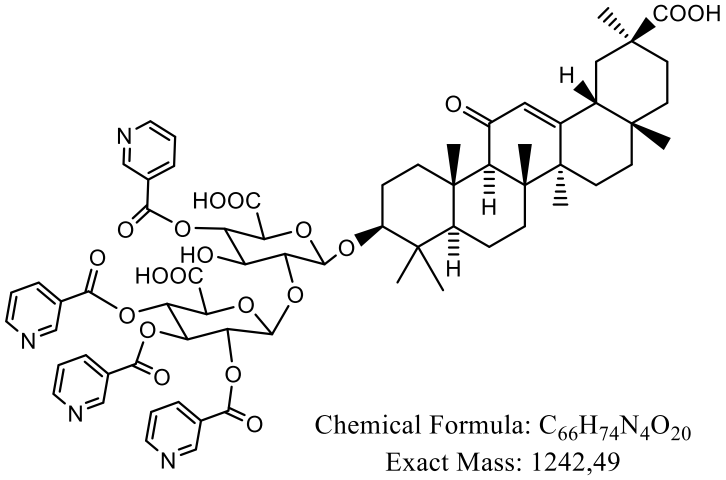

Chemical Formula: $C_{66}H_{74}N_{4}O_{20}$
Exact Mass: 1242,49
Molecular Weight: 1243,33
m/z: 1242.49 (100.0%), 1243.49 (71.4%), 1244.50 (25.1%), 1245.50 (5.8%), 1244.49 (4.1%), 1245.50 (2.9%), 1243.49 (1.5%), 1244.49 (1.1%), 1246.50 (1.0%)

Figure S11.

Primary data on antiviral activity against the virus SARS-CoV-2.

Inhibitory activity of chemical compounds Glicivir and Remdesivir against SARS-CoV-2 virus strains:
Fig. S12, S13 - hCoV-19/Australia/VIC01/2020 (B);
Fig. S13, S14 - hCoV-19/Russia/MOS-2512/2020 (B 1.1.7.);
Fig. S15, S16 - hCoV-19/Russia/PSK-2804/2021 (B 1.617.2).
Figures were made with SOFTmax PRO 4.0 software using the 4-parametric method of analysis.
Concentrations of chemical compounds are given in µg/ml.

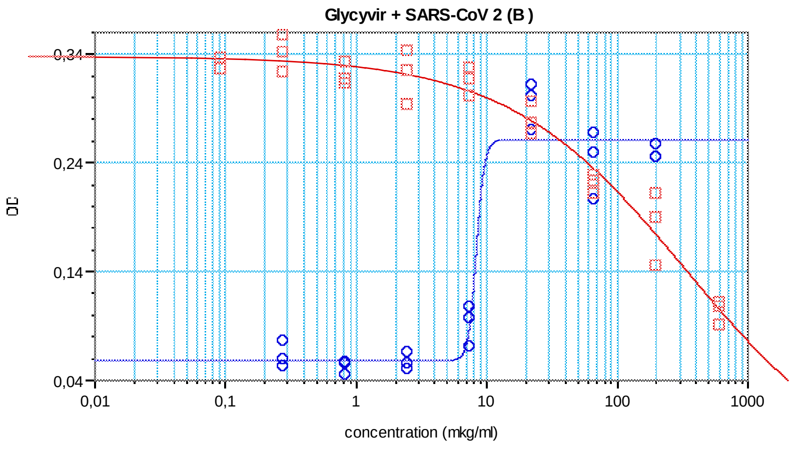

y = ((A - D)/(1 + (x/C)^B)) + D:	A	B	C	D	R^2
activity (activity: Concentration vs Values)	0,058	14,019	8,304	0,262	0,958
toxity (toxity: Concentration vs Values)	0,338	0,633	334,47	-0,055	0,957

Figure S12.

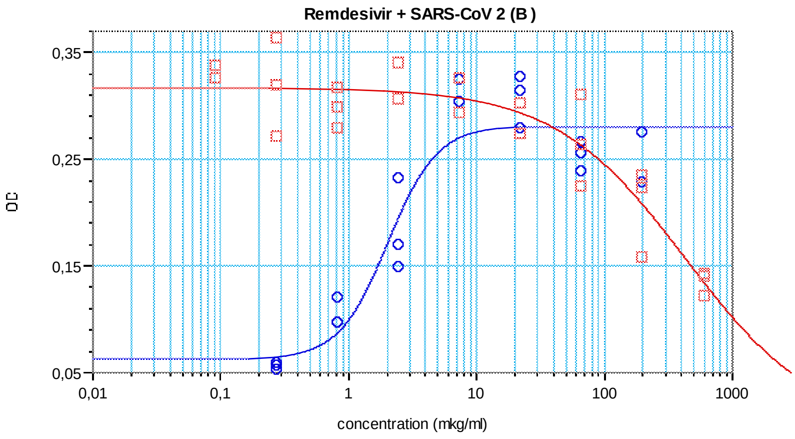

y = ((A - D)/(1 + (x/C)^B)) + D:	A	B	C	D	R^2
activity (activity: Concentration vs Values)	0,062	2,257	2,016	0,281	0,91
toxity (toxity: Concentration vs Values)	0,317	0,847	428,695	-0,004	0,854

Figure S13.

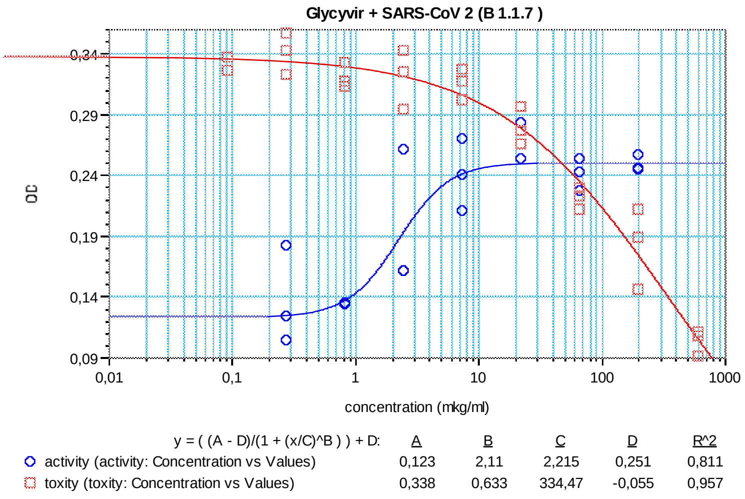

Figure S14.

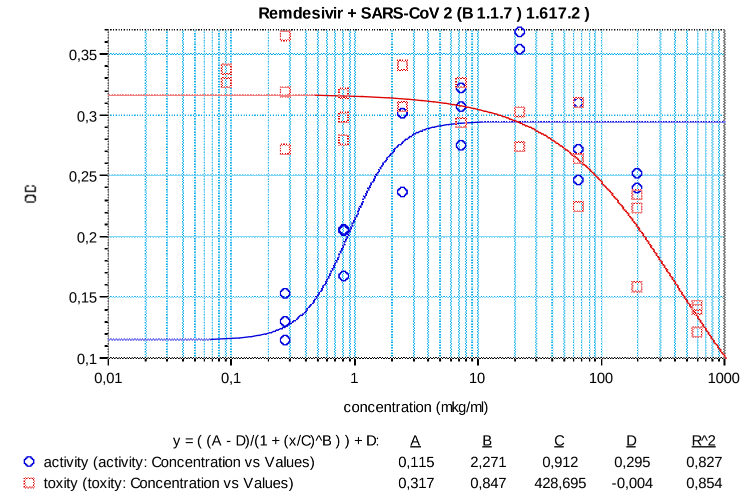

Figure S15.

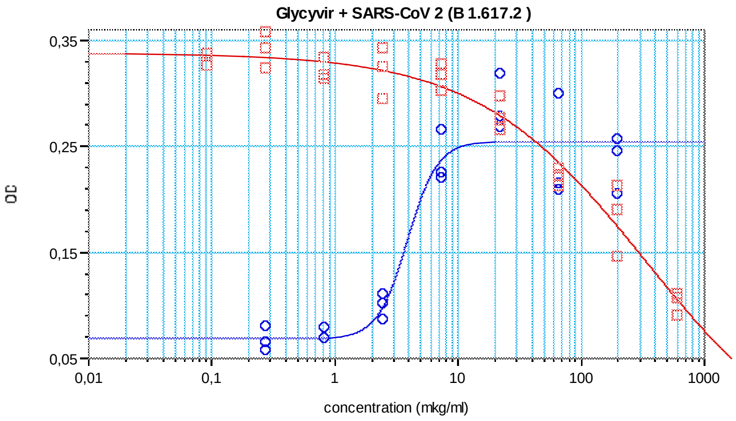

y = ((A - D)/(1 + (x/C)^B)) + D:	A	B	C	D	R^2
activity (activity: Concentration vs Values)	0,068	3,567	3,866	0,255	0,92
toxity (toxity: Concentration vs Values)	0,338	0,633	334,47	-0,055	0,957

Figure S16.

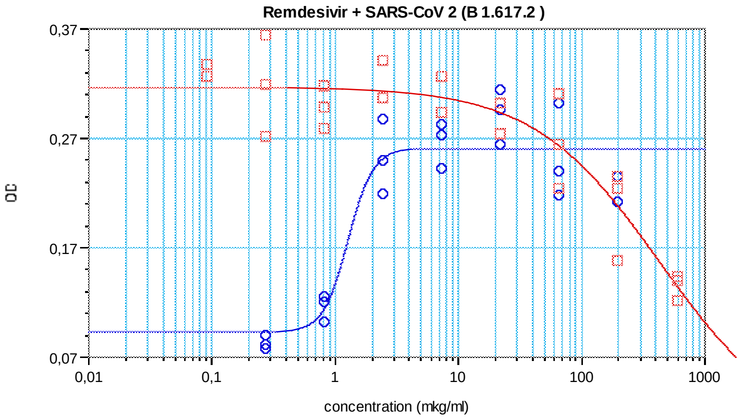

y = ((A - D)/(1 + (x/C)^B)) + D:	A	B	C	D	R^2
activity (activity: Concentration vs Values)	0,093	4,29	1,269	0,261	0,891
toxity (toxity: Concentration vs Values)	0,317	0,847	428,695	-0,004	0,854

Figure S17.
